# Linguistic Markers in At-Risk Mental States Using Natural Language Processing: A Systematic Review

**DOI:** 10.3390/healthcare14080999

**Published:** 2026-04-10

**Authors:** Yuhan Zhang, Alba Carrió, Julia Sevilla-Llewellyn-Jones, Enrique Gutiérrez, Ana Calvo, Jose-Blas Navarro, Ana Barajas

**Affiliations:** 1 Department of Clinical and Health Psychology, Autonomous University of Barcelona, 08193 Bellaterra, Spain; yuhan.zhang@uab.cat (Y.Z.); albacarriopsicologa@gmail.com (A.C.); 2 Instituto de Psiquiatría y Salud Mental, Instituto de Investigación (IdISSC), Hospital Clínico San Carlos, 28040 Madrid, Spain; julia.sevilla@salud.madrid.org; 3 Department of Applied Mathematics for Information and Communication Technologies, Higher Technical School of Computer Systems Engineering, Polytechnic University of Madrid, 28040 Madrid, Spain; enrique.gutierrez.alvarez@upm.es; 4 MIT Linq, Massachusetts Institute of Technology, Cambridge, MA 02139-4307, USA; 5 Personality, Assessment and Clinical Psychology Department, School of Psychology, Universidad Complutense de Madrid, 28040 Madrid, Spain; anabcalv@ucm.es; 6 Department of Psychobiology and Methodology of Health Sciences, Autonomous University of Barcelona, 08193 Bellaterra, Spain; 7 Serra Húnter Programme, Government of Catalonia, 08002 Barcelona, Spain

**Keywords:** at-risk mental state, psychosis, linguistic markers, natural language processing, artificial intelligence

## Abstract

**Highlights:**

**What are the main findings?**

- NLP markers, particularly reduced semantic coherence, lower syntactic complexity, and diminished referential cohesion, significantly differentiate individuals with at-risk mental states (ARMS) from healthy controls.
- Automated linguistic indicators can predict the transition to psychosis with high accuracy levels (ranging from 79% to 100%).

**What are the implications of the main findings?**

- NLP serves as an objective, non-invasive, and cost-effective tool that complements traditional semi-structured interviews in clinical settings.
- The integration of automated speech analysis into early detection protocols could enhance prognostic specificity, enabling more timely and targeted preventive interventions for individuals at clinical high risk.

**Abstract:**

**Background/Objectives**: In recent years, research on psychosis has increasingly focused on prevention, aiming to implement early interventions that mitigate or reduce its impact. Within this framework, the analysis of linguistic markers in individuals with at-risk mental states (ARMS) has proven valuable for identifying those at risk and predicting psychosis onset. Artificial intelligence tools, particularly natural language processing (NLP), have emerged as effective resources for detecting these language-based indicators. This study aims to synthesize the existing scientific evidence on linguistic markers analyzed through NLP techniques in individuals with ARMS. **Methods**: A systematic review following the PRISMA 2020 protocol was conducted. Three databases (PubMed, PsycInfo, and Scopus) were searched for published articles from their inception to October 2025. Rayyan software was used to manage references and article downloads. Out of ninety initial search results, fifteen studies involving 1313 participants from diverse groups were included in the review. **Results**: The findings indicated that alterations in semantic coherence, syntactic complexity, referential cohesion, and speech/content poverty differentiated ARMS individuals from healthy controls. Several of these markers, analyzed with NLP methods, predicted the onset of psychosis with accuracy levels ranging from 79% to 100%, although these findings should be interpreted with caution due to the significant methodological heterogeneity and variability in sample sizes across the included studies. **Conclusions**: NLP techniques offer a powerful approach for detecting language alterations that distinguish ARMS individuals and provide meaningful predictions of psychosis onset, highlighting their potential as a complement to traditional clinical assessments for early identification and prevention.

## 1. Introduction

In recent decades, the scientific community has witnessed a significant shift toward the prevention of psychotic disorders, given their status as a leading cause of long-term disability in young adults [1]. As functional alterations across multiple domains can be identified during the prodromal phase, clinicians have adopted a preventive approach aimed at reducing the probability of transition to psychosis diagnosis or, at minimum, attenuating the severity of the disease [2,3]. Early detection is clinically relevant not only for identifying individuals at high risk but also for the timely implementation of interventions that maximize the degree of recovery and reduce the duration of untreated psychosis (DUP) [4,5,6]. Absence or delay in treatment often leads to first-episode psychosis (FEP), which is associated with marked declines in education, employment, and social functioning [7], underscoring the need to refine detection strategies toward the earliest possible stages of risk [8].

To identify individuals prior to the emergence of overt symptomatology, frameworks such as the Ultra High Risk (UHR) [9] and Clinical High Risk (CHR) [10] have been developed. The concept of an at-risk mental state (ARMS) is a broad clinical framework used to identify individuals in this vulnerable period. However, ARMS is not a single diagnostic entity; rather, it represents a heterogeneous group characterized by various subthreshold mental health conditions and pre-existing vulnerabilities. The development of ARMS is often preceded by a combination of genetic load (family history of psychotic disorders), premorbid personality traits (such as high levels of schizotypy), and environmental stressors, including childhood trauma or significant social isolation. Furthermore, individuals with ARMS often present with comorbid subthreshold mood disorders, such as depression and anxiety, which interact with emergent cognitive impairments to facilitate the onset of risk symptoms [11]. Semi-structured interviews like the Comprehensive Assessment of At-Risk Mental States (CAARMS) [12] and the Structured Interview for Prodromal Symptoms (SIPS/SOPS) [10] remain the gold standard for ARMS diagnosis, but their predictive specificity is notably low. Previous longitudinal studies have shown that only 29% to 36% of subjects identified as being at clinical high-risk develop psychosis within a three-year follow-up period [13]. This diagnostic gap suggests that relying solely on traditional clinical interviews may be insufficient for accurate prognosis. Consequently, there is an urgent need for objective complementary markers that can improve the sensitivity and specificity of current assessment techniques.

Language abnormalities are increasingly recognized as important predictive markers for the onset of psychosis, with recent studies investigating these alterations as indicators of underlying formal thought disorders [14]. Evidence has shown that specific linguistic features, including pronoun usage, syntactic complexity, speech cohesion, and lexical density, can differ significantly between individuals with ARMS and healthy controls [3,15].

Various artificial intelligence methods have been explored to identify ARMS. Machine learning has been applied to analyze neuroimaging data (MRI/fMRI) to detect structural brain changes, as well as to electronic health records (EHR) and digital phenotyping from smartphone sensors to track behavioral patterns. While these approaches offer objective metrics, they often require expensive infrastructure, invasive procedures, or face significant data privacy hurdles, limiting their applied use in research and, most notably, in clinical environments. The most widely used artificial intelligence method for identifying linguistic markers in individuals with ARMS is natural language processing (NLP) [16], owing to its ability to analyze large volumes of text both automatically and accurately. NLP leverages machine learning algorithms to detect abnormal patterns in speech and writing by examining syntactic, semantic, and pragmatic features. For example, previous studies have shown that NLP methods were able to detect that compared to healthy controls, people with schizophrenia used more pronouns but fewer adverbs, adjectives, and determiners in speech. Additionally, NLP techniques have demonstrated the capacity to identify diagnosis-specific patterns that reflect the distinct etiological mechanisms of different disorders. For instance, while both schizophrenia and first-episode bipolar disorder may present with disorganized speech, NLP-based research indicates that schizophrenia is often characterized by a more pronounced breakdown in semantic coherence and structural simplicity (e.g., incomplete words), reflecting core neurocognitive deficits. In contrast, linguistic alterations in bipolar disorder typically manifest as increased verbal productivity and a ‘flight of ideas’ driven by affective dysregulation [17]. These findings suggest that NLP can capture the unique ‘linguistic signature’ of each disorder, providing insights into their differing underlying psychotic processes [17]. NLP methods were also able to detect that those with FEP demonstrated higher semantic similarity in speech [18]. The NLP approach has also proven effective in predicting and detecting the transition to psychosis [19]. Furthermore, automated NLP techniques are capable of identifying complex and subtle language patterns that may go unnoticed by clinicians assessing ARMS using traditional methods. The markers detected through this approach can uncover anomalies that, according to several studies, help predict the transition to psychosis with substantially higher accuracy than that achieved using traditional clinical interview methods [15,19,20].

The aim of this systematic review is to synthesize the existing scientific evidence regarding linguistic markers in individuals with ARMS using NLP techniques, addressing two distinct clinical questions: (1) the diagnostic identification of the ARMS state (discriminating at-risk individuals from healthy controls) and (2) the prognostic prediction of psychosis transition (discriminating between those who later convert to psychosis and those who do not). Additionally, this review examines how linguistic markers identified in individuals with ARMS compared with those observed in first-episode psychosis (FEP) to evaluate the existence of a linguistic continuum from subthreshold risk to overt illness.

To ensure conceptual clarity, this review uses ‘at-risk mental states (ARMS)’ as the standard clinical term for the population under study, and ‘transition’ to refer to the longitudinal onset of psychosis. Regarding the linguistic markers analyzed via NLP, detailed definitions and clinical examples for each of these linguistic constructs are provided in Table A1 (Appendix A). In addition, a broad and inclusive definition of FEP is applied, encompassing individuals within approximately the first 2–5 years of psychotic symptom onset, regardless of prior treatment exposure, while acknowledging that operational definitions vary across studies. This approach is consistent with the current recommendations for early psychosis research and facilitates meaningful integration of studies with heterogeneous inclusion criteria.

## 2. Materials and Methods

### 2.1. Search Strategy

This review was conducted in line with the Preferred Reporting Items for Systematic Reviews and Meta-Analyses statement (available upon request) [21]. The PRISMA checklist is presented in the Appendix A.

A systematic search of published studies was conducted using the following databases: PubMed, Scopus and PsycInfo, from database inception to October 2025. This broad timeframe was chosen to ensure a comprehensive historical mapping of the field, allowing for the identification of core linguistic markers that remain consistent regardless of the evolving computational power. The three databases were selected to prioritize studies with a high level of clinical evidence. While engineering-focused repositories as IEEE Xplore exist, the current review aimed to synthesize findings derived from validated clinical cohorts and standardized psychiatric assessments, for which medical and psychological databases provide the most relevant and rigorously peer-reviewed literature.

The search focused on linguistic markers in ARMS populations analyzed using NLP techniques. Only articles published in English were considered, and no restrictions were applied regarding the year of publication. Additional articles were identified by manually searching the references of retrieved articles and relevant reviews. The general search strategy for each database was designed by combining keywords, the search term repository (thesaurus, MeSH), and Boolean operators (AND, OR). The keywords used in the search strategy for each database are shown in Table 1.

**Figure 1:**
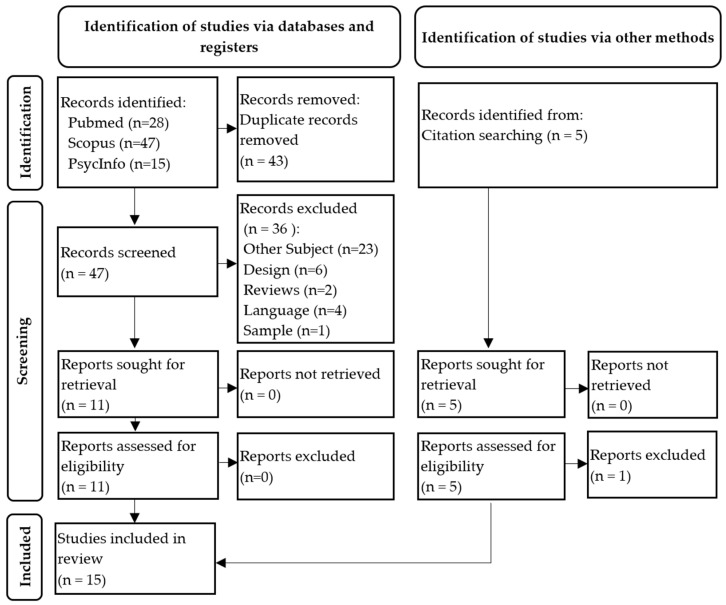
Flowchart of the study selection process for the systematic review.

The results were imported into the Rayyan platform [22] for duplicate detection and removal, as well as for title, abstract and full-text screening of relevant studies (see Figure 1).

### 2.2. Eligibility Criteria

Articles published in English that examined linguistic markers in ARMS populations using NLP and artificial intelligence techniques were included. No restrictions on the sociodemographic characteristics of the analyzed samples were applied. Any research design was considered (observational or experimental, cross-sectional or longitudinal), but single-case studies, literature reviews, systematic reviews, and meta-analyses were excluded. No restrictions were applied based on the year of publication.

### 2.3. Data Extraction

The first co-authors (Y.Z. and A.C.) were responsible for data extraction from all included articles, and any discrepancies were resolved through discussion with two of the co-authors (J.-B.N. and A.B.). The extracted data encompassed the following: (i) first author and year of publication, (ii) target population and sample size, (iii) ARMS diagnostic tool, (iv) language collection method, (v) language analysis technique, (vi) analyzed language variables, and (vii) main findings. In this review, language collection methods refer to the approaches used to obtain speech samples for subsequent analysis, whereas language analysis techniques denote the tools and/or applications employed to process, examine, and extract features from these samples, including any preprocessing procedures.

### 2.4. Quality Assessment and Risk of Bias

The methodological quality and risk of bias of the included studies were independently assessed by two reviewers (J.-B.N. and Y.Z.). Any discrepancies were resolved through consensus or by consulting a third senior reviewer (A.B.). The JBI Critical Appraisal Tools for Analytical Cross-Sectional Studies for quantitative papers was used. Studies were evaluated across several domains, including inclusion criteria clarity, setting description, validity and reliability of the linguistic measurements (NLP techniques), identification of confounding factors (such as age, education, or medication), and appropriateness of statistical analysis. No studies were excluded based on quality scores.

## 3. Results

To ensure a structured and comprehensive synthesis of the evidence, the section is organized into three thematic levels. First, we present the study selection process and the results of the methodological quality and risk of bias assessment. Second, we provide an overview of the study characteristics, detailing the clinical populations and diagnostic tools (SIPS/CAARMS) used across the 15 included studies. Finally, we move from descriptive reporting to a thematic synthesis of linguistic markers, grouping the findings into four core domains: semantic coherence, syntactic complexity, referential cohesion, and linguistic poverty.

### 3.1. Study Selection

A total of 90 articles were identified through searches of the three databases and manual screening of bibliographic references. After removing duplicates, the first author (Y.Z.) independently reviewed the titles and abstracts of the 47 remaining publications, selecting 11 for full-text review (see Figure 1). Subsequently, using the Rayyan platform [22], two independent reviewers (J.-B.N. and A.B.) agreed to include the 11 articles during the full-text screening phase. A search of the bibliographic references of the 11 articles was then done, which revealed 5 potentially relevant articles, from which only one was excluded because of the language. Ultimately, 15 articles were included in this systematic review.

### 3.2. Methodological Quality and Risk of Bias Assessment of Included Studies

The formal quality assessment indicated that the majority of the studies (n = 13; 86.6%) exhibited high methodological quality, with a low risk of bias. Most studies provided detailed descriptions of the clinical participants (SIPS/SOPS or CAARMS criteria) and utilized robust NLP pipelines. The most common limitation identified was the small sample size in some studies and the lack of explicit strategies to deal with all potential confounders (e.g., the effect of antipsychotic medication on speech production). Despite these limitations, the statistical rigor—specifically the use of cross-validation and permutation testing—was deemed appropriate for the respective sample sizes. A detailed breakdown of the quality assessment for each study is provided in Table A2 (see Appendix A).

### 3.3. Study Characteristics

The list of included studies and their main characteristics is presented in Table 2. The sample sizes ranged from 24 to 93 ARMS participants, with only one study exceeding this range, including 167 subjects [23]. Seven of the fifteen studies also included groups with first-episode psychosis (FEP) in addition to control groups, while only three studies focused exclusively on ARMS participants. Regarding ARMS diagnosis, eleven studies utilized the Structured Interview for Psychosis (SIPS)—usually in conjunction with the Scale of Prodromal Symptoms (SOPS)—, whereas five studies employed the Comprehensive Assessment of At-Risk Mental States (CAARMS).

Various methods were employed to collect speech samples, including open-ended narrative interviews and the Thematic Apperception Test (TAT) being the most used. A range of computational methods was applied for language analysis. The Natural Language Toolkit (NLTK) was the most frequently utilized, while other methods, such as Latent Semantic Analysis (LSA), Specific Speech Part Tagging (POS-Tag), S-BERT Bidirectional Sentence Encoder, and Speech Graph Software 2.0, were also employed in several studies, albeit less frequently. The language variables most frequently analyzed were grouped into semantic coherence, syntactic complexity, referential cohesion, and formal thought disorders, with poverty of speech and poverty of content being the most extensively studied (see Table A1, Appendix A for definitions of these variables).

An initial consideration regarding the 15 reviewed articles is the distinction between those that exclusively employ computational language analysis techniques (13 out of 15) and hybrid studies that incorporate manual expert assessment into the computational outputs [26,27].

The main findings indicated the following linguistic markers as the most prominent in individuals with ARMS:

Semantic coherence. Three articles reported reduced semantic coherence in individuals with ARMS compared to healthy controls [19,26,28]. Moreover, Bilgrami et al. [26] found a correlation between semantic coherence and formal positive thinking disorders (tangentiality, circumstantiality, and derailment), as assessed using the Thought, Language, and Communication Assessment Scale (TLC). In another study, semantic coherence with syntactic complexity and speech/content poverty in ARMS participants was associated with measures of brain structure and functional connectivity [25]. In contrast, Dalal et al. [29] suggested most ARMS individuals demonstrated semantic coherence aligned with a typical linguistic profile along with the controls. Additionally, Kizilay et al. [30] reported contradictory findings, showing higher semantic coherence in ARMS individuals than in the control group. Nevertheless, the authors of this study noted that they developed a machine learning model based on NLP-derived features that achieved 79.6% accuracy in distinguishing ARMS individuals from controls.

Syntactic complexity. Three studies reported reduced syntactic complexity in individuals with ARMS compared to control subjects [19,26,30]. Another study found that syntactic complexity was correlated with negative symptoms and appeared sensitive to prodromal symptoms in ARMS participants [25]. Similarly, Bilgrami et al. [26] reported that syntactic complexity was associated with formal negative thought disorder (speech/content poverty), as assessed using the Thought, Language and Communication Assessment Scale (TLC).

Referential cohesion. One study reported reduced referential cohesion in individuals with ARMS compared to control subjects [24]. Additionally, three studies found that referential cohesion was lower in individuals who later developed psychosis compared to the ARMS group [14,16].

Poverty of speech/content. One study reported higher levels of speech/content poverty in individuals with ARMS compared to controls [26]. Furthermore, other study found that speech poverty was higher in ARMS individuals who subsequently transitioned to psychosis than in those who did not [20].

### 3.4. Linguistic Markers as Predictors of Psychosis

Three of the included studies specifically examined the predictive capacity of certain language variables for psychosis. Bedi et al. [15] demonstrated that semantic coherence, syntactic complexity, and speech/content poverty jointly contributed to accurately predicting the onset of psychosis. Corcoran et al. [19] reported that reduced semantic coherence and syntactic complexity, assessed through the use of possessive pronouns, predicted psychosis with 79% accuracy. Finally, Rezaii et al. [20] found that speech/content poverty, along with the use of words relative to voices and sounds mentioned during the SIPS/SOPS interview, collectively predicted psychosis with 90% accuracy. All three studies employed the Natural Language Toolkit (NLTK), one of the most used NLP tools for language analysis in peer-reviewed studies.

Additionally, retrospective analyses of speech samples in other studies indicated that referential cohesion [14,16] and speech/content poverty [20] were also significant predictors of psychosis.

### 3.5. Additional Language Measures Beyond NLP in ARMS Research

Two of the included studies examined additional language measures complementary to those obtained through NLP. Baklund et al. [27] compared the validity of NLP measures with basic self-disturbance (BSD) scores, noting that BSD measures are qualitatively different and can serve as additional clinical risk markers in pre-psychotic stages. Similarly, Srivastava [23] analyzed the semantic similarity between the natural speech of ARMS individuals and items from the Inventory of Psychotic-Like Anomalous Self-Experiences (IPASE), which assess anomalous self-experiences. The results showed higher semantic similarity in ARMS individuals compared to controls. These complementary measures provide further insights into linguistic alterations, enriching the predictive and clinical information obtained from NLP analyses.

## 4. Discussion

This systematic review has synthesized the current scientific evidence on the analysis of linguistic markers in the ARMS population. A key insight from this research is the increasing integration of advanced technologies, particularly NLP methods and machine learning approaches, into clinical psychopathology assessment over the last decade. While various computational methods were identified, including the use of NLP libraries like the Natural Language Toolkit (NLTK) for feature extraction, the application of these features within machine learning models suggests promising, though still emerging, avenues for identifying language patterns characteristic of the ARMS population. These patterns hold potential for early identification and prediction of transition to psychosis. A recent systematic review by García-Molina et al. also evaluated the application of NLP to speech analysis applied to ARMS groups, focusing primarily on the diagnostic and prognostic performance of these models (e.g., accuracy, sensitivity, specificity, and AUC-ROC) [33]. Although both reviews examine NLP studies in at-risk populations, Garcia-Molina et al. primarily evaluated diagnostic and transition accuracy, organizing their synthesis around predictive performance. In contrast, our review places equal emphasis on the linguistic features extracted by these models, systematically examining patterns in semantic, syntactic, and discourse-level markers to clarify the language characteristics associated with psychosis risk. Thus, while the prior review focuses on how well NLP models perform, the present work seeks to elucidate what linguistic signals underlie these predictions.

The primary linguistic markers consistently highlighted across the reviewed studies, derived largely from NLP-based analyses, include alterations in semantic coherence, syntactic complexity, referential cohesion, and the manifestation of formal thought disorders such as poverty of speech/content. Beyond these primary NLP-derived markers, this review also identified studies exploring complementary language measures in ARMS individuals. For instance, Baklund et al. [27] investigated BSD linguistic markers, suggesting they may offer qualitatively different insights, while Srivastava et al. [23] examined the semantic similarity between natural speech and IPASE items. These studies suggest that a multi-modal approach to language analysis, potentially combining computational NLP features with qualitatively distinct measures, might offer a more comprehensive understanding of language alterations in the ARMS population. The discussion of how these complementary measures relate to, or could be integrated with, NLP-derived features warrants further exploration in future research.

The reviewed literature predominantly focused on linguistic markers capable of distinguishing the ARMS population from healthy controls and, in some instances, from individuals experiencing an FEP. Several studies employed retrospective analyses of speech samples from individuals who later transitioned to psychosis (ARMS+) versus those who did not (ARMS−), thereby identifying potential differentiating linguistic features. Others specifically investigated the predictive capacity of certain linguistic markers, reporting varying degrees of accuracy. Addressing the question of which linguistic markers best distinguish the ARMS population from healthy individuals, this review suggests that reduced semantic coherence and diminished syntactic complexity are among the most frequently reported trends, although the findings across studies remain heterogeneous. Secondary to these, poverty of speech/content and reduced referential cohesion also emerged as relevant differentiators.

Semantic coherence, in particular, was the most frequently investigated language characteristic within the included studies of this review. The predominant finding of reduced semantic coherence in ARMS individuals suggests its potential as a robust linguistic marker. This aligns with theoretical perspectives where the analysis of semantic coherence is considered a strategy to identify subclinical manifestations of formal thought disorders, such as derailment, in the ARMS population [34] (See Table A1, Appendix A). Further supporting this, Bilgrami et al. [26] reported an association between semantic coherence and positive thought disorders, as assessed by the TLC. Notably, they also observed that coherence was negatively correlated with negative symptoms, likely reflecting the reduced linguistic complexity and shorter utterances characteristic of individuals with prominent negative symptomatology, which may constrain opportunities for derailment. Given the relative consistency of these findings, semantic coherence appears to be a critical feature to consider in the development of any automated language analysis tool for ARMS assessment. However, the operationalization of “semantic coherence” can vary significantly depending on the NLP techniques employed (e.g., latent semantic analysis vs. graph-based methods vs. transformer-based embeddings), and the impact of this methodological heterogeneity on the robustness of this finding requires careful consideration.

Reduced syntactic complexity also emerged as a robust linguistic marker distinguishing individuals with ARMS from controls. From a psychopathological perspective, studies by Haas et al. [25] and Bilgrami et al. [26] demonstrated its association with negative symptoms. Hence, reduced syntactic complexity may represent a promising linguistic feature for the early identification of subthreshold manifestations of psychosis, particularly within the negative symptom domain, although further validation is required.

Regarding linguistic markers predictive of psychosis, a degree of consensus was observed across studies evaluating semantic coherence, syntactic complexity, and speech/content poverty. These studies reported predictive accuracies ranging from 79% to 100%, though substantial methodological diversity and varying cohort sizes across studies necessitate a cautious interpretation of these outcomes [15,19,20]. It is crucial to interpret these high accuracy figures with caution, considering the often small sample sizes and methodological heterogeneity in the primary studies, which can affect model generalizability. Referential cohesion also showed potential as a predictor, with reduced cohesion observed in ARMS individuals who later transitioned to psychosis (ARMS+) [14,16]. The use of NLP toolkits like NLTK was common across several predictive studies [15,19,20], though the specific features extracted and the machine learning algorithms subsequently applied likely varied, underscoring the need for better methodological transparency and standardization in future predictive modelling research.

Additionally, although direct comparisons between ARMS and FEP were limited across the included studies, the available evidence suggests partial overlap in key linguistic alterations, particularly reduced semantic coherence and syntactic complexity. These shared features may support the hypothesis of a linguistic continuum from subthreshold risk states to overt psychotic illness. However, the extent to which these alterations reflect a progressive worsening versus qualitatively distinct phenomena remains unclear. Further longitudinal and cross-diagnostic studies are needed to better characterize the trajectory and specificity of these linguistic changes across the psychosis spectrum.

A notable limitation of most of the included studies was their small sample sizes. This is particularly pertinent for studies employing machine learning techniques, where small datasets can lead to model overfitting and limit the generalizability of findings, necessitating replication in larger independent cohorts. Furthermore, the inclusion of participants on antipsychotic medication in some studies introduces a potential confounder, as medication effects could influence language production and thus the observed results.

Methodological heterogeneity across analytical approaches poses a significant challenge. To ascertain the predictive utility of language variables for psychosis transition, studies have employed diverse statistical analyses and machine learning models. This methodological variability, while reflective of an exploratory phase in the field, complicates the direct comparison of findings and the formulation of generalized conclusions regarding the most effective computational strategies. The path from identifying “linguistic markers” to establishing them as validated clinical “biomarkers” is long and requires rigorous testing for reliability, specificity, and clinical utility, which is currently underexplored. In addition, it is important to note that the study of linguistic markers in the ARMS population is still very recent, and consequently, the available scientific evidence remains limited. Furthermore, only articles published in English were included, meaning that potentially relevant evidence in other languages may have been overlooked.

These findings may have relevant clinical implications for the detection and prevention of psychosis, as well as for the treatment of the ARMS population. Linguistic markers can reveal the presence of subclinical symptoms in early stages before the full onset of clinical symptoms, allowing intervention to be applied to individuals who are at potential risk of transitioning to psychosis in order to prevent or mitigate its impact. The application of AI and machine learning allows for the analysis of large linguistic datasets and the detection of subtle alterations that might be missed by human evaluators, thereby enhancing the accuracy and efficiency of identifying these markers. Such tools could complement existing clinical assessments, potentially improving the diagnostic precision. However, the practical implementation of this methodology into routine clinical settings will require addressing challenges such as data privacy, integration with electronic health records, and clinician training.

Some critical considerations should be considered when interpreting the conclusions of this work. First, it is necessary to consider the evolution of NLP technology. The included studies span from early models based on Latent Semantic Analysis (LSA) and part-of-speech tagging [15] to modern transformer-based models (e.g., BERT, SBERT) [23,26,30]. While earlier statistical methods established foundational markers like semantic coherence, they often lacked the context-awareness of current architectures. Interestingly, predictive accuracies have remained high across eras; however, modern models offer superior robustness and handle the inherent complexity of natural language more effectively, potentially reducing the risk of overfitting seen in smaller earlier cohorts. A second critical consideration refers to whether the reported deficits in linguistic markers are specific to ARMS or reflect a general signature of cognitive decline. Similar NLP-derived alterations, such as reduced syntactic complexity and semantic density, have been documented in neurodegenerative conditions like Alzheimer’s and Parkinson’s diseases [35,36]. However, important qualitative differences exist. In Alzheimer’s, linguistic impairment is typically driven by semantic memory loss and lexical retrieval deficits (often resulting in ‘empty speech’), whereas in ARMS, the markers are primarily associated with the breakdown of logical thought trajectories and formal thought disorder. While Parkinson’s disease also affects verbal fluency, it is often characterized by motor–speech impairments (dysarthria) and executive dysfunction rather than the semantic incoherence found in the psychosis spectrum. Future research using NLP should focus on multi-diagnostic comparisons to further refine the specificity of these ‘digital biotypes’. A third and final consideration is about the number of published studies applying natural language processing to clinical high-risk (CHR) populations, which remains limited and concentrated within a small number of research groups. This relative scarcity also reflects the early developmental stage of the field. Importantly, large-scale initiatives such as the Accelerating Medicines Program in Schizophrenia have recently made available a substantial multilingual corpus of de-identified clinical transcripts through the NIH National Data Archive, including longitudinal outcome data and extracted linguistic features. These resources are expected to generate a rapid increase in NLP-based studies of CHR populations in the coming years.

In this context, the current review is intended not as a definitive synthesis of a mature literature but as a conceptual and methodological framework to support the next phase of research. By critically evaluating existing linguistic metrics, theoretical assumptions, and sources of heterogeneity across studies, this review aims to provide a foundation for interpreting future multi-center large-scale findings, facilitating cross-study comparability, and guiding the development of clinically meaningful linguistic biomarkers. As increasingly complex NLP approaches—including large language models—are applied to expansive datasets, such grounding may be particularly important to ensure that advances in predictive performance remain aligned with psychopathological theory and clinical relevance.

For future research, several avenues are apparent. There is a pressing need for studies with larger and more diverse samples to validate the current findings and to train more robust machine learning models. Standardization of both language data collection methods and NLP/machine learning analytical pipelines is crucial for improving the comparability and reliability of results. This could involve developing benchmark datasets and encouraging comparative studies of different computational approaches on the same data. Furthermore, longitudinal studies are essential to more definitively establish the predictive validity of linguistic markers for psychosis transition. Investigating the interpretability of complex machine learning models used in this domain is also important to ensure clinical acceptance and understanding. While the results of this systematic review warrant cautious interpretation, they provide a foundational basis for directing further investigation into linguistic markers that show significant promise for identifying ARMS individuals and predicting the onset of psychosis. Lastly, emerging studies have begun to examine the emotional valence of language in ARMS populations. For instance, recent findings indicate that narratives produced by ARMS group contain a higher proportion of incongruent negative emotional words compared to healthy controls [31], suggesting affective disturbances in spontaneous speech. Future research could expand on this line of inquiry by systematically quantifying positive and negative emotional word use and by exploring how such affective–linguistic patterns interact with established markers such as semantic coherence and syntactic complexity.

## 5. Conclusions

This systematic review highlights the potential of linguistic marker analysis in the ARMS population to identify subclinical symptoms in prodromal phases of psychosis using different NLP methods. It has been observed that the use of this type of techniques has made it possible to detect alterations in language, such as reduced semantic coherence, reduced syntactic complexity and poor speech/content, which may help distinguish individuals with ARMS from healthy controls and contribute to the prediction of psychosis onset. These findings point to a promising and potentially fruitful direction for improving clinical evaluation and early detection. Likewise, the study of linguistic markers using NLP techniques also contributes to the development of solid theoretical models on the relationship between language alterations and subthreshold symptoms in the ARMS population. NLP methods provide automated high-throughput analysis but should be regarded as complementary to gold-standard clinician assessments. Traditional expert evaluations, though time-intensive, offer the high clinical interpretability that current black-box computational models often lack.

While the findings are promising, the limitations identified in the included studies (e.g., small sample sizes, methodological heterogeneity) warrant a cautious interpretation of the results. Nonetheless, the evidence reviewed provides a valuable foundation to inform and guide more rigorous future research in this rapidly evolving field.

## Figures and Tables

**Table 1:** Specific search strategies.

Databases	Search Expression
Medline	(“ultra high risk”[Title/Abstract] OR “clinical high risk”[Title/Abstract] OR CHR[Title/Abstract] OR UHR[Title/Abstract] OR “risk for psychosis”[Title/Abstract] OR “at-risk mental state”[Title/Abstract] OR “at-risk for psychosis”[Title/Abstract]) AND (“language analysis”[Title/Abstract] OR “language markers”[Title/Abstract] OR “linguistic markers”[Title/Abstract] OR “natural language processing”[Title/Abstract])
Scopus	(TITLE-ABS-KEY(“ultra high risk”) OR TITLE-ABS-KEY(“clinical high risk”) OR TITLE-ABS-KEY(CHR) OR TITLE-ABS-KEY(UHR) OR TITLE-ABS-KEY(“risk for psychosis”) OR TITLE-ABS-KEY(“at-risk mental state”) OR TITLE-ABS-KEY(“at-risk for psychosis”)) AND (TITLE-ABS-KEY(“language analysis”) OR TITLE-ABS-KEY(“language markers”) OR TITLE-ABS-KEY(“linguistic markers”) OR TITLE-ABS-KEY(“natural language processing”))
PsycInfo	(tiab(“ultra high risk”) OR tiab(“clinical high risk”) OR tiab(CHR) OR tiab(UHR) OR tiab(“risk for psychosis”) OR tiab(“at-risk mental state”) OR tiab(“at-risk for psychosis”)) AND (tiab(“language analysis”) OR tiab(“language markers”) OR tiab(“linguistic markers”) OR tiab(“natural language processing”))

**Table 2:** Studies analyzing linguistic markers in ARMS.

Reference	Groups and N	ARMS Diagnostic Instrument	Language Collection Method	Language Analysis Technique	Language Variables Analyzed	Main Results
Bedi (2015) [15]	ARMS = 34	SIPS/SOPS	Open narrative interviews.	Natural Language Toolkit (NLTK). Latent Semantic Analysis (LSA).	· Semantic coherence. · Syntactic complexity: use of determiners, poverty of speech/content.	· Semantic coherence, normalized use of determiners, and speech/content poverty: they predict psychosis with 100% accuracy.
Corcoran (2018) [19]	ARMS = 93 CG = 21 FEP = 16	SIPS/SOPS	“Story Game”. Open narrative interviews.	Natural Language Toolkit (NLTK). Latent Semantic Analysis (LSA). Tagging specific parts of speech (POS-Tag) with Penn Treebank. Machine learning classifier.	· Semantic coherence. · Syntactic complexity: use of possessive pronouns.	· Semantic coherence: ARMS < CG. · Semantic coherence variance: ARMS > CG. · Possessive pronouns: ARMS < CG. · They predict psychosis with 79% accuracy.
Gupta (2018) [24]	ARMS = 41 CG = 43	SIPS/SOPS	Narrative description task written using the Boston Cookie Theft Image.	Coh-Metrix 3.0.	· Referential cohesion.	· Referential cohesion: ARMS < CG.
Rezaii (2019) [20]	ARMS = 40	SIPS/SOPS	SIPS/SOPS interview recordings.	Stanford PCFG parser. WordNetLemmatizer module of the Natural Language Toolkit (NLTK).	· Poverty of speech/content. · Syntactic complexity: use of determiners. · Probability of saying words “Voice, sound, song or loud”.	· Poverty of speech/content: ARMS+ > ARMS−. · Use of words over voices and sounds: ARMS+ > ARMS−. · They predict psychosis with 90% accuracy.
Haas (2020) [25]	ARMS = 46 CG = 22	SIPS/SOPS	Open narrative interviews.	Natural Language Toolkit (NLTK). Latent Semantic Analysis (LSA).	· Poverty of speech/content. · Semantic coherence. · Syntactic complexity.	· Syntactic complexity: correlates with negative symptoms and seems sensitive to prodromal symptoms in ARMS individuals. · Speech/content poverty, semantic coherence, and syntactic complexity: correlated with measures of brain structure and functional connectivity in ARMS individuals.
Spencer (2021) [14]	ARMS = 24 FEP = 16 CG = 13	CAARMS	Thematic Apperception Test (TAT).	Speech Graph Software.	· Referential cohesion: graph-based analysis of connectivity of speech.	· Referential cohesion: ARMS+ < ARMS−.
Morgan (2021) [16]	ARMS = 25 FEP = 16 CG = 13	CAARMS	Thematic Apperception Test (TAT). Speech Comprehension Test (DCT). Interview on any topic.	Measurements using NLP. Embedding Google News, Word2vec and SIF model words. Latent Semantic Analysis (LSA). Calculus cosine similarity between all possible sentence pairs. Pre-trained conference resolution model. Speech Graph Software.	· Semantic coherence. · Tangentiality. · Relationship between the discourse and the theme of the DCT. · Repetition. · Number of ambiguous pronouns. · Referential cohesion: graphic connectivity of speech.	· Semantic coherence: ARMS < CG.
Bilgrami (2022) [26]	ARMS = 60 CG = 27	SIPS/SOPS	Open qualitative interviews. Thought, Language and Communication Assessment Scale (TLC).	Natural Language Toolkit (NLTK). Tagging of specific parts of speech: (POS-Tag). Transformer Bidirectional Encoder Representation (BERT).	· FTA measures: Elements of thought disorder+: tangentiality, circumstantiality, derailment. Elements of thought disorder: poverty of speech/content. · NLP Measurements: Semantic coherence, syntactic complexity (poverty of speech/content and use of determiner pronouns).	· Semantic coherence: ARMS < CG. Correlated with TLC + thought disorder (tangentiality, circumstantiality, and derailment). · Syntactic complexity (poverty of speech/content and use of determinant pronouns): ARMS < CG. Correlated with TLC thought disorder (speech/content poverty).
Baklund (2023) [27]	ARMS and BSD = 30	PQ16 SIPS/SOPS	Semi-structured interviews from the Anomalous Self-Experience Examination Manual (EASE).	An adapted form of Interpretive Phenomenological Analysis (IPA) based on a linguistic conceptual framework and the concept of basic self-disturbance (BSD).	· Distinctive and prominent words related to language. · Irregular use of prepositions related to place and location. · Personal pronouns. · Use of conjunctions and metaphors. · Idiosyncratic use of adjectives and perceptual modalities.	· BSD Linguistic Markers: Appear qualitatively different from the linguistic markers analyzed in studies with ARMS individuals.
Srivastava (2023) [23]	ARMS = 167 FEP = 89 CG = 170	SIPS/SOPS CAARMS	Open qualitative interviews. IPASE.	Natural Language Toolkit (NLTK). S-BERT bidirectional sentence encoder.	· Semantic similarity of natural speech with anomalies of ego experiences assessed by IPASE.	· Semantic similarity between natural speech and IPASE (anomalous experiences of the self): ARMS > FEP > CG.
Nettekoven (2023) [28]	ARMS = 24 FEP = 16 CG = 13	CAARMS	Thematic Apperception Test (TAT). Speech Comprehension Test (DCT).	Speech networks transcribed in Python 2.0 (netts). Speech syntactic graphics.	· Connected components of the netts-generated semantic speech network.	· Semantic networks of speech: CG > ARMS > FEP.
Dalal (2025) [29]	ARMS = 18 PSY = 18 FEP = 72 CG = 39	SIPS	Thought and Language Index (TLI).	Cluster Analysis with R package Nbclust.	· Choice of words or lexical variables. · Structure of utterances or syntax variables. · Semantic cohesion.	Three cluster solution: · Largest cluster with typical linguistic profile included most CG and the majority of ARMS and PSY. · Cluster with high semantic similarity in word choices with less perceptual words, lower cohesion and analytical structure mostly contained FEP. · Last cluster with more perceptual but less cognitive/emotional word classes, simpler syntactic structure, and a lack of sufficient reference to prior information has more PSY.
Kizilay (2024) [30]	ARMS = 62 CG = 45	SIPS/SOPS	Thematic Apperception Test (TAT).	Natural Language Toolkit (NLTK). S-BERT bidirectional sentence encoder. Machine Learning Classification: Forest classifier with Python scikit-learn.	· Semantic coherence. · Image and text similarity. · Tangentiality. · Generic characteristics and specific parts of speech (POS).	· Semantic coherence and use of adjectives: ARMS > CG. · Similarity between image and text: ARMS < CG. · Adverbs, conjunctions and pronouns in the first person: ARMS < CG. · Machine learning model based on NLP features achieved an accuracy of 79.6% in the discriminative capacity of ARMS vs. CG individuals.
Mota (2025) [31]	ARMS = 42 CG = 29	SIPS, the Prodromal Questionnaire, the PCA scale	The Happy Thoughts protocol.	Speech Graph Software. Linguistic Inquiry Word Count.	· Largest connected component. · Largest strongly connected component. · Proportion of positive and negative emotional words.	Narratives with a higher proportion of “incongruent” negative emotional words: ARMS > CG.
Kim-Dufor (2025) [32]	ARMS = 45 CG = 15 FEP = 8	CAARMS	Open narrative interviews.	Latent semantic analysis (LSA). Supervised machine learning model XGBoost.	· Lexical richness, diversity, density. · Syntactic complexity. · Semantic coherence. · Speech fluency.	Intersubjective LSA minimum: CG < FEP, ARMS < FEP. Subjective LSA minimum: ARMS < FEP.

BSD: basic self-disturbance. CAARMS: Comprehensive Assessment of At-Risk Mental States. ARMS+: ARMS patients who developed psychosis. ARMS−: ARMS patients who did not develop psychosis. CG: control group. IPA: Interpretative Phenomenological Analysis. IPASE: Inventory of Psychotic-Like Anomalous Self-Experiences. NLTK: Natural Language Toolkit. PCFG: Probabilistic Context-Free Grammar. FEP: First Psychotic Episode Group. NLP: natural language processing. PSY: patients with psychosis. POS: part of speech. S-BERT: Sentence Bidirectional Encoder Representation from Text. SIPS: Structured interview for psychosis. SOPS: Scale of Prodromal Symptoms. TLC: Thought, Language and Communication Assessment Scale.

## Data Availability

No new data were created or analyzed in this study.

## Abbreviations

The following abbreviations are used in this manuscript:

ARMS	Individuals with At-Risk Mental States
NLP	Natural Language Processing
PRISMA	Preferred Reporting Items for Systematic Reviews and Meta-Analyses
FEP	First-Episode Psychosis
CHR	Clinical High Risk for Psychosis
DUP	Duration of Untreated Psychosis
UHR	Ultra High Risk

## Appendix A

**Table A1:** Definition and examples of the language variables analyzed.

Language Variables Analyzed	Definition	Examples
Reduced referential cohesion	Referential cohesion involves the use of a pronoun, demonstrative, definite or comparative article to refer to people or objects previously mentioned. In a loosely cohesive reference, the speaker uses a pronoun, demonstrative, definite or comparative article to refer to a person or object that has not been previously mentioned, which can confuse the listener. Similarly, the listener is confused if the speaker makes an ambiguous reference when using a referent that can be applied to more than one person or object [37].	Therapist: What did John do? Patient: John went to the park. John took him. John played with his dog. In this sentence with reduced referential cohesion, there is a lack of connectors that refer to the person mentioned above, repeating the name “John”, instead of a pronoun. In addition, a reference is made to an object that has not been mentioned before “him” (dog). A cohesive answer would be: “John went to the park with his dog and played with him.”
Reduced semantic coherence	Logical organization of the meaning of discourse through interrelated linguistic structures. The lack of semantic coherence makes it difficult to understand the discourse and integrate the meaning of sentences [38].	Therapist: What are you going to do today to eat?” The lack of semantic coherence will be revealed with a response like: “Macaroons. This afternoon I will go shopping for tobacco. I think my brother called me yesterday.” In this response, you can see how the topic of conversation changes abruptly, going from answering what is asked to talking about anything else, without there being a logical continuity in the discourse.
Reduced syntactic complexity	Syntactic complexity is defined as the degree of elaboration and diversity in the grammatical structures used in the discourse, reflected in the subordination, coordination and use of modifying elements that enrich the sentences [39]. The use of pronouns, determiners, conjunctions, adverbs, verbs, etc., reflect the level of syntactic complexity of a discourse or narrative.	Therapist: Why did the children get wet? Patient: “They left without an umbrella.” The first sentence shows a reduced level of syntactic complexity compared to an answer such as the following: “Although it was raining heavily, the children, who had been waiting all day, went out to the park to play, carrying raincoats and boots, but forgot the umbrella.”
Poverty of speech/content	The measures of speech poverty (decrease in the quantity of speech) and content poverty (decrease in the quality of thought) have been unified under the concept of speech poverty/content; also included under this category are measures that refer to sentence length or semantic density.	
Speech poverty	There is a decrease in the amount of spontaneous speech, with the answers being brief, not very fluent, fragmentary, vague and not elaborated. It is rare for additional information to be provided that has not been specifically asked. The patient may not even speak if not asked and answer only in monosyllables (yes, no, etc.), and some questions may remain unanswered [40].	Therapist: “What did you do yesterday?” Patient: “I was home.” Therapist: “And what else did you do at home?” Patient: “Nothing.”
Poverty of content	There is a decrease in the quality of thought. The language is adequate in quantity (verbal fluency is preserved), and the answers are sufficiently long, but they provide little information. Language tends to be vague, repetitive, imprecise, abstract and stereotyped. One can speak fluently without giving the appropriate information to answer the question asked [9].	Therapist: “How have you been feeling lately?” Patient: “I’ve been… not bad. Everything is… well, only… Nothing is the same as before. That’s all… like this. As always.”
Tangentiality	A disorder of the course of thought that is characterized by the inability to associate goal-directed thoughts. They respond obliquely to what is asked and lose the thread of the conversation. There is a lack of relationship between the question and the answer given. The patient gets lost in ramblings; an answer related to the general theme is given, but they do not answer the question asked. The final goal is not reached [40].	Therapist: “How are you feeling today?” A tangential response would be like, “Well, the weather is changing a lot lately. It’s cold in the mornings and then hot in the afternoons. Climate change is a real problem, and I think it affects animals as well. Yesterday I saw a documentary about that…”
Repetition (persevering thinking)	It consists of the repetition of the same answer to different questions; the patient is practically unable to change the answers. In it, words, phrases or ideas are repeated, out of context. One continuously dwells on the same concepts and gives persistent answers despite the fact that new questions or stimuli may appear [40].	Therapist: “What did you do this morning?” Patient: “I went to the market. There were people in the market, many people. The market is always full. In the market they sell fruits. I like the market. The market is close to my house. Do you like the market?”
Circumstantiality	A disorder of the course of thought that is characterized by language with excessive and redundant information. Difficulty in selecting ideas, such that it cannot be discerned between what is essential and what is accessory. There is a loss of the ability to direct thought towards a goal. Excessive, unnecessary, irrelevant details are incorporated, with multiple paragraphs and clarifying comments and with difficulties in arriving at the final idea [40].	Therapist: “Did you go to the grocery store today?” Patient: “Yes, but first I had to look for the keys, which I always leave on the table, although sometimes I put them in the drawer, because I also keep other things there such as invoices, important papers, and once I found a blue pen there… Oh yes, I went to the supermarket.”
Derailment	A language disorder characterized by the interruption of the logical connection between ideas and the general sense of direction of thought. Constant sliding from one topic to another. Individual sentences can be clear and meaningful, although there is a lack of an adequate connection between phrases or ideas. The resulting language may be non-cohesive, and the final content of the speech may not be related to the question asked at the beginning [40].	Therapist: “What did you do today?” Patient: “Today I had toast for breakfast, because I like it a lot. The cows give milk, but I think the grass they eat is green. Yesterday I saw a red car that was going very fast. The color red has always reminded me of traffic lights… have you ever seen a broken traffic light?”
Literal use of metaphors	A blurred boundary between the symbolic and literal meaning of metaphors has been identified in ARMS subjects [28].	“It’s like seeing through the eyes of a body I’ve bought or a robot,” “my thoughts are on autopilot, as if my true thoughts have been put aside,” “it’s like everything is organized, like in a theater.”

**Table A2:** Quality assessment of included studies using JBI Critical Appraisal Tools.

Reference	Q1	Q2	Q3	Q4	Q5	Q6	Q7	Q8	Overall Quality
Bedi (2015) [15]	Y	Y	Y	Y	U	U	Y	Y	Moderate/High
Corcoran (2018) [19]	Y	Y	Y	Y	Y	Y	Y	Y	High
Gupta (2018) [24]	Y	Y	Y	Y	Y	Y	Y	Y	High
Rezaii (2019) [20]	Y	Y	Y	Y	Y	Y	Y	Y	High
Haas (2020) [25]	Y	Y	Y	Y	Y	Y	Y	Y	High
Spencer (2021) [14]	Y	Y	Y	Y	Y	Y	Y	Y	High
Morgan (2021) [16]	Y	Y	Y	Y	Y	Y	Y	Y	High
Bilgrami (2022) [26]	Y	Y	Y	Y	Y	Y	Y	Y	High
Baklund (2023) [27]	Y	Y	Y	Y	Y	Y	Y	Y	High
Srivastava (2023) [23]	Y	Y	Y	Y	Y	Y	Y	Y	High
Nettekoven (2023) [28]	Y	Y	Y	Y	Y	Y	Y	Y	High
Dalal (2025) [29]	Y	Y	Y	Y	Y	Y	Y	Y	High
Kizilay (2024) [30]	Y	Y	Y	Y	Y	Y	Y	Y	High
Mota (2025) [31]	Y	Y	Y	Y	Y	Y	Y	Y	High
Kim-Dufor (2025) [32]	Y	Y	Y	Y	U	U	Y	Y	Moderate/High

Y: Yes; N: No; U: Unclear; Q1: inclusion criteria; Q2: subjects and setting; Q3: valid exposure/marker measurement; Q4: objective condition criteria; Q5: confounders identified; Q6: strategies for confounders; Q7: valid outcome measurement; Q8: statistical analysis.
